# Building Relationships, Forming Collaborations: Lessons Learned From an Unconference Seeking to Cultivate Solutions in Healthcare

**DOI:** 10.1111/hex.70021

**Published:** 2024-09-12

**Authors:** Brenda M. Y. Leung, Helen Kelley, Angie Nikoleychuk, Gabrielle Kirk, Fatemeh Salehi Shahrabi, Victoria Hecker, Nolan Schaaf

**Affiliations:** ^1^ Faculty of Health Sciences University of Lethbridge Lethbridge Alberta Canada; ^2^ Dhillon School of Business University of Lethbridge Lethbridge Alberta Canada; ^3^ Department of Psychology University of Lethbridge Lethbridge Alberta Canada; ^4^ Department of Nutrition Services, Population and Public Health Alberta Health Services Lethbridge Alberta Canada; ^5^ Chinook Primary Care Network Lethbridge Alberta Canada

**Keywords:** collaborations, healthcare, meeting, transdisciplinary, Unconference

## Abstract

**Introduction:**

Calls for a ‘major rethinking’ of the delivery of healthcare services are echoed across Canada as the healthcare crisis continues. Proposed strategies to address the challenges of this crisis include: a transdisciplinary approach that is patient‐focused and community‐based; a representative team composed of patients, caregivers, healthcare providers, decision makers and policymakers; and authentic collaboration among stakeholder groups throughout the research cycle.

**Objective:**

This study aimed to enable community members to take on a leading role in building capacity and to provide a space for discourse among diverse groups while respecting community wisdom, values and priorities.

**Methods:**

The Collaborative Health Research Institute of Southern Alberta (CHRISA) organized a participant‐oriented Unconference event to address the factors contributing to the healthcare crisis in Alberta, Canada. An Unconference is a participant‐oriented meeting where the attendees nominate the topics, agree on the agenda and lead the sessions. This article describes the Unconference programme and presents the findings from a thematic analysis of the discussion notes from breakout sessions, feedback from participants (i.e., lessons learned) and pragmatic recommendations for future Unconference events.

**Results:**

Findings from sessions included the following: (1) identifying the ‘wicked’ problems, (2) the factors/causes contributing to each problem (i.e., contributors) and (3) potential multifaceted solutions or ideas to remedy the problem. Lessons learned from the postevent evaluation resulted in six recommendations for organizing future Unconferences.

**Conclusion:**

The CHRISA Unconference achieved its goals by providing a venue for attendees to connect, engage and network on topics of interest, explore new ways of addressing challenges in healthcare and serve as a foundation for future initiatives and collaborations in healthcare research and practice.

**Patient or Public Contribution:**

The Unconference was attended by community members who identify as patients, frontline workers, programme administrators and representatives of public organizations and agencies. Participants contributed to breakout session discussions, provided feedback on the Unconference and offered recommendations for future events. The co‐authors are service users, people with lived experience or those work in the healthcare setting; they have been involved in data collection, analysis and interpretation, and contributed to this report.

## 1 Introduction

The healthcare system in Canada is facing constant challenges, notably the disruptive burdens of the COVID‐19 pandemic, growing cases of chronic diseases, long‐term demands on limited resources and services and the complexity of meeting the needs of an ageing population. The deteriorating state of the healthcare system across Canada has been a constant topic in the news [1], with headlines such as ‘health‐care delivery needs a major rethink’ [2] in the face of the realities for many people, including the shortage of frontline staff, long wait times and lack of access to emergency care, primary care and specialists. Much of the discourse has questioned the sustainability of the healthcare system, with the pressures of greater demand for services in healthcare [3], increasing responsibilities assigned to fewer healthcare professionals [4] and fewer fiscal and human resources [5].

To address many of these issues in healthcare, impactful research requires a team approach encompassing patients, caregivers, frontline workers, practitioners, decision makers and other partners outside of healthcare [6]. That is, authentic collaborations are created before conceptualizing ideas for research, then at all stages, from generating research questions to translating findings into action. Healthcare research aimed at improving overall health, quality of life and well‐being of citizens requires a transdisciplinary approach that is patient‐focused and community‐based. Both Alberta and the Federal Government of Canada have made collaborative, patient‐oriented and community‐engaged research a funding priority.

The Collaborative Health Research Institute of Southern Alberta (CHRISA) was created in response to the growing need for transdisciplinary, patient‐focused and community‐based health research across the lifespan. Situated within the University of Lethbridge, CHRISA aims to facilitate collaborative relationships between researchers, trainees and the communities of patients, clinicians/practitioners and decision makers to pursue research that addresses critical questions related to individual, family and community health.

As a ‘hub’ to facilitate impactful health research, CHRISA planned an ‘Unconference’ event to bring together partners to discuss grassroots initiatives. An Unconference is a participant‐oriented meeting where the attendees nominate topics, agree on the agenda and lead the sessions. The goal was to enable community members to take a leading role in building capacity and provide a space for discourse among diverse groups while respecting community wisdom, values and priorities. As recommended by Swann et al., ‘an essential component of community collaboration is allowing time and space to nurture connections between researchers and community partners; this includes having informal, agenda‐less conversations’ [7]. This article describes the goals, processes and participant perspectives and outcomes of the CHRISA Unconference.

### 1.1 Unconference Setting and Context

CHRISA organized an innovative conference, the ‘CHRISA Unconference— Cultivating Solutions in Healthcare’ in May 2023. *Unconferences* are a nontraditional form of professional activity defined by the absence of many conventional conference structures [8]. The CHRISA Unconference was designed to (1) support engagement among diverse groups and organizations, (2) enable collaboration with a broad range of community partners and (3) create a platform for developing transdisciplinary research. The CHRISA Unconference provided a venue for bringing disciplines, sectors and people together to think ‘outside the box’ in addressing the issues in healthcare. The Unconference format allowed attendees from diverse disciplines and backgrounds to work collaboratively on topics of common interest [9]. The CHRISA Unconference prioritized conversation over presentation, examining topics relevant to attendees and emphasizing contributions from participants.

## 2 Materials and Methods

### 2.1 Description of the CHRISA Unconference

Promotion materials for the Unconference were distributed to healthcare professionals, university faculty members, community health agencies, community leaders and other partners. Posters were emailed to the CHRISA membership list, who were asked to distribute them to their network of contacts (a snowballing technique). Leading community health agencies were identified and extended personal email invitations to the event and/or were contacted directly. Posters were also distributed throughout the university and CHRISA social media platforms (e.g., Facebook, Instagram and Twitter) to reach a broader audience. Registration for the 2‐day event was free. CHRISA provided snacks, beverages and lunches for all registrants.

Registration was free of charge using the Qualtrics programme, and a total of 105 registrations were received. At the time of registration, registrants were asked to list their topics of interest for the breakout sessions and whether they would like to facilitate a breakout session.

#### 2.1.1 Facilitators Training

Facilitators were provided with written material, a ‘facilitator's guide’ with information on strategies and tips for effective facilitation and answers to frequently asked questions. We also hosted two facilitator training webinars to provide an overview of the objectives of the Unconference and discuss their roles and responsibilities as facilitators. We recommended facilitators use the Liberating Structure methods by Lipmanowicz and McCandless to guide the discussion and to consider what they would like to achieve [10, 11, 12] from the Unconference. Liberating structures are techniques to foster engagement and interaction in groups [13] and have been shown to optimize participation in health professionals and education systems [10, 12]. Both the facilitator guide and the training webinar explained the Liberating Structures, which consisted of 33 practical methods (e.g., wicked questions, what I need from you, improv prototyping, and shift and share) that facilitators could choose to lead their breakout sessions. The advantages of Liberating Structures are simple, easy to learn and enhance coordination to generate lively participation resulting in profound discussions and outcomes. The Liberating Structure methods aligned with the goals of the Unconference to ‘think outside the box’ and ‘find new ways’ of doing things to help address the healthcare crisis.

### 2.2 Unconference Format

The CHRISA Unconference was a 2‐day event. The objective of day 1 was to enable attendees to establish rapport over common topics of interest and engage in identifying the issues and ideas towards solutions to form collaborations. The day consisted of two keynote presentations and 20 breakout sessions. In the morning, Dave Price and Teri Price presented on ‘How Can We Enable Better Teamwork?’, which provided a patient voice to their personal experiences of the healthcare system and the need to improve quality patient safety. In the afternoon, Wendy Mulse and Brandy Old presented ‘Crash Course on Design Thinking’ strategies to develop innovation in healthcare. Design thinking is a systematic innovation process that prioritizes empathy for end‐users to develop comprehensive and effective solutions [14]. After each keynote presentation, attendees attended breakout sessions offered throughout the day, which consisted of two 5‐concurrent sessions in the morning, and another two 5‐concurrent sessions in the afternoon. At the end of day 1, attendees were asked to vote on the topics for day 2. A total of eight topics (i.e., sessions) were selected from the 20 sessions offered on the first day.

The objective for day 2 was to deepen the discussions of the topics selected from day 1, with the goal of developing ideas for addressing the issues and generating a plan for collaborators to work beyond the Unconference. The schedule for day 2 included two keynote speakers: Dr. Don MacIntyre spoke on ‘Indigenization, the Unstory’, the concept of bringing Indigenization to healthcare; and Dr. Aaron Low, South Zone Medical Director, utilized a Q&A format to discuss current pressing issues and needs in healthcare in southern Alberta. See Appendix A for the Unconference Agenda.

Sessions were led by one or two facilitators using Liberating Structures and Design Thinking to guide the discussions and using the following discussion points to form collaborations:

- Identify the pressing issue/problem in need of urgent attention:
  o Probe question: What is the issue/concern/problem in healthcare you'd like to change?
- Engage with attendees to collaborate on addressing the issues/problems:
  o Probe question: Who wants to be involved in this change or these changes?
- Generate a plan for long‐term impact. For example, develop a proposal, seek out grants or expertize/partners, find resources or test a concept as the next steps following the Unconference.

### 2.3 Breakout Session Notes Review

Each breakout session had one to two student volunteers as notetakers. Notetakers recorded discussions using a standardized Google Doc form to record the main points of the discussions in real time. The Google Doc sections included the following: (1) session title and facilitators, (2) discussion points, (3) keywords and (4) action items. The Google Doc form was live and displayed on an overhead screen in the main meeting room where participants could review and update the document in real time during the day.

In the subsequent weeks following the Unconference, three of the authors (Brenda M. Y. Leung, Gabrielle Kirk, and Fatemeh Salehi Shahrabi) reviewed the session notes to collate the key discussion points. Each person reviewed the session notes independently using thematic analysis to determine common concepts within the notes. Each reviewer read the notes multiple times (5–10) and derived meaning from the text to categorize the concepts coming out of the data. After all the notes were reviewed and analyzed, the three reviewers met to discuss their results. In the discussion, commonalities and differences in the findings were explored and deliberated to arrive at a consensus on the final concepts.

### 2.4 Lessons Learned—Post‐Unconference Evaluation

After the Unconference, an evaluation was sent to attendees. The evaluation provided attendees' perspectives on their experience at the Unconference, such as meeting other attendees from different areas/backgrounds and forming collaborations. The evaluation also asked about their input on the strengths and weaknesses of the Unconference approach and recommendations for improvement in the future.

## 3 Results

### 3.1 Summary of Breakout Session Discussion Notes

Common categories were identified from the breakout session discussion notes and grouped by similarity of concepts: (1) the ‘wicked’ problem, (2) the factors/causes contributing to the problem (i.e., contributors) and (3) potential multifaceted solutions or ideas to remedy the problem. One wicked problem identified was the challenge to patients in navigating the healthcare system with contributing factors/causes of siloed departments limited in communication channels leading to misinformation of service availability and access, the duplication of services which is further compounded by a shortage of healthcare professionals and services. Attendees proposed some potential multifaceted solutions, which include the following: improving communication channels among providers and integrating a more holistic approach to enable continuity of services that are patient oriented and community based. The second wicked problem identified was the issue of disparity in access and quality of care resulting in health inequity. The contributors to the problem included the following: stigmatization and discrimination of vulnerable groups due to ethnicity/race, sexual orientation, age and financial need, as well as the urban/rural divide and people from Indigenous communities. To resolve this wicked problem, recommendations included policies for culturally competent care that are patient focused, better accessibility for vulnerable groups and those with financial constraints. The third wicked problems underscore the systematic barriers to service delivery, which are associated with the absence of comprehensive and integrated multimodal care, the shortage of healthcare providers and the complex bureaucracy impeding service delivery. Solutions proposed were as follows: enabling better collaboration and integration across departments and organizations, increasing the use of digital health systems and more long‐term sustainability funding.

Table 1 summarizes the key points of the breakout session discussions, including a summary of the three wicked problems, their contributing factors and potential solutions.

**Table 1:** Key findings from breakout session discussions.

Wicked problem	Contributors (root causes and factors)	Potential solutions
Navigating the healthcare system is difficult for patients	Healthcare departments are siloed: The separation and lack of coordination between organizations and departments increases the complexity for patients to navigate the system and creates fragmented services.	Collaboration and engagement between healthcare providers: opportunities for collaboration and developing strong communication channels for healthcare providers will enhance coordination. 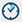
	Limited communication: Information sharing between healthcare providers is hindered by inadequate communication channels, resulting in patient care gaps. Patients can also be left out of necessary communications related to their care.	Integrated and holistic approaches: Silos and streamlined services can be broken down through integrated and holistic approaches to care. It will help provide comprehensive care that addresses the overall well‐being of patients. 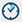
	Duplication of services: Because of ineffective communication between service providers and healthcare departments, services may be duplicated, such as unnecessary tests or procedures.	Community‐based integration of services: Bringing together healthcare providers, community organizations and partners to ensure a patient‐centred approach to care. 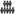 , 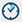 , 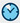
	Misinformation: Patients often receive a lack of clear or accurate information regarding available healthcare services, eligibility criteria and referral processes. This can result in confusion and delays in accessing needed care.	Implement care/service navigators or similar supports to help patients navigate services that are informed and understandable for patients. 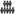 , 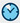
	Shortage of healthcare professionals and services: Accessing relevant and timely care is difficult with shortages of healthcare professionals and programmes. For example, the shortage of family physicians is a barrier for patients accessing specialist care as family physicians are needed for referrals.	Create policy and programmes to increase the training of medical professionals and expand the scope of practice for para‐health professions. 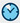
Health inequality perpetuates disparities in access, quality of care and outcomes	Stigmatization and discrimination: Stigmatization and discrimination based on race, ethnicity, sex, sexual orientation and age can lead to unequal treatment and limited access to health services and quality care.	Culturally Competent care: Training and education for healthcare providers can enhance cultural competencies, enabling them to deliver respectful and personalized care. 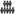 , 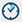
	Urban versus rural: The availability of healthcare services varies between urban and rural settings. Individuals living in rural communities have reduced access to local services and have increased barriers to accessing services in urban areas.	Increase accessibility of health services: Removing barriers to healthcare access is critical for reducing health inequalities—for example, language or geographical barriers. 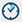 , 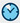
	Jurisdictional barriers for Indigenous people: Coordinating care between on‐reserve and off‐reserve healthcare providers can create challenges due to jurisdictional boundaries. The division of authority between different levels of government can be complex and lead to jurisdictional ambiguity.	Patient perspectives: Patients’ perspectives can highlight their unique challenges and be crucial for developing patient‐centred care models and policies. 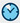 , 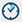
	Financial strain: Income and health are directly related to each other. Individuals facing financial strain may struggle to access or afford necessary healthcare services and medications.	Reducing the impact of financial strain: Addressing financial strain in clinical practice can help remove barriers to accessing health services and increase health outcomes. This can be through affordable healthcare coverage, subsidies and financial assistance programmes. 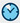 , 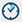
Systematic barriers to the delivery of healthcare services	Inadequate multimodal approaches: An absence of comprehensive and integrated approaches, including a lack of access to different therapeutic modalities, can result in suboptimal health outcomes.	Collaboration and integration of different organizations: Fostering partnerships and collaborations between various healthcare sectors can optimize resource utilization, share expertize and improve healthcare delivery. Models such as Collective Impact can serve as a template to support system integration activities [15]. 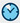 , 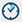 , 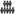
	Healthcare professional shortages: An insufficient number of healthcare professionals, such as physicians, nurses and allied healthcare workers, can lead to an increase in wait times and increased workloads and limit access to care.	Digitalization and health systems: Investing in digital health infrastructure, such as electronic medical records and telehealth platforms, can streamline healthcare delivery, improve communication and enhance access to services. 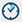 , 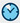
	Bureaucracy: Complex administrative processes, excessive paperwork and bureaucratic hurdles can impede service delivery and lead to delays.	Adequate funding: Adequately allocate financial resources to ensure the availability of equipment, supplies and staff, as well as to support research, innovation and quality improvement. 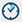 , 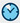
	Limited digitalization: Limited adoption of digital health technologies can hinder communication, coordination and delivery of healthcare services.	Explore virtual or e‐health technology, especially for patients living in rural areas.

*Note:* 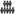 represents advocacy; 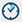 represents short‐term action; and 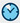 represents long‐term action.

### 3.2 Summary of Evaluation Responses

Thirty responses to the post‐Unconference evaluation survey were received. The lessons learned from responses to the questions are summarized as follows:

*What is your field of expertize/interest?*

Participants came from diverse professional and work backgrounds. The fields of expertize/interest provided by respondents were grouped by health disciplines, health areas and community areas (Table 2).

**Table 2:** Expertize and interests of respondents.

Health disciplines	Health areas	Community areas
Psychology Human services Prevention and interventions Health leadership/management Public health Environmental health Health promotion Not‐for‐profit sector Patient education Health literacy Social work	Addiction Mental health Harm reduction Primary care Nutrition Integrated health/social care Seniors’ health Palliative care nursing Sexual health	Supported housing Healthy communities Community advocacy Food security Community health services Health and wellness from a community lens Advocating for vulnerable Indigenous populations

*What did the Unconference achieve for you?*

Most participants reported they had the opportunity to establish new connections and develop their social networks during the event. They also had the chance to understand and appreciate different perspectives, questions and struggles of others. Participants acknowledged enthusiasm for the new ideas related to health promotion and addressing unique assets and healthcare needs in rural communities. Furthermore, the Unconference sparked interesting concepts surrounding greater community engagement and information sharing. Some of the participants acknowledged that they enjoyed the breakout sessions and the opportunity to exchange ideas, whereas some felt that not much tangible output was generated. For many participants, the CHRISA Unconference met its goals to bring diverse people together to enable connections and networking to occur, and further allow the sharing of information and insights.

*What aspects of the Unconference would you improve?*

Some participants suggested having a problem presentation on day 1 followed by discussions and solutions on day 2. Others expressed their preference for condensing the event into one single day. Some participants felt that the unstructured nature of the conference might deter them from recommending it to their colleagues. However, others enjoyed the second day of the Unconference and suggested that elected officials and decision makers should have been invited to this event. Furthermore, considering the rural perspective, timing the event to avoid conflicting with important agricultural activities such as seeding was suggested. Some sessions lacked discussion and actionable takeaways, whereas other unguided sessions left groups unsure of where to begin. Overall, participants expressed the need for focused topics, networking opportunities and structured discussions to enhance the Unconference experience.

*What would you like to see as the next steps post‐Unconference?*

Most participants acknowledged their interest in connecting with other attendees for future work or collaborations. Participants expressed that having findings and recommendations with an action plan for moving forward would be beneficial. Additionally, sharing information collected through the sessions with those in a position to effect change would be valuable. Implementing solutions and continuing discussions to bring about meaningful change, building connections and organizing focus groups to explore previous topics further would be valuable steps to take post‐Unconference.

## 4 Discussion

The CHRISA Unconference provided the venue for attendees from different professional and personal backgrounds to connect, engage and network on topics of interest to explore new ways of addressing the challenges in healthcare. The CHRISA Unconference followed the recommendations of Witteman et al.^6^ to hold early in‐person meetings, with introductions focused on motivation, offer appropriate orientation for everyone, ask for and recognize diverse contributions, seek the input of all members and facilitate good communication.

The post‐Unconference evaluation revealed the participants' positive experiences, including the opportunity to establish new connections, understand different perspectives and generate innovative ideas. Responses to the evaluation demonstrated that the Unconference achieved its goals: (1) attended by participants with a diverse range of expertize and interests and (2) provided attendees with the opportunities to engage and network.

There was also room for improvement, as suggested by respondents. One area for improvement was the Unconference format as some participants felt it was too unstructured, lacked actionable takeaways and needed more guidance for a productive discussion. Given most facilitators were unfamiliar with the nature of the Unconference format, and despite training provided to the facilitators (i.e., a facilitator's guide and webinar training session), additional training and instructions may help with more effective breakout sessions. Depending on the session, some topics were quite narrow, such as vaping, whereas others were broader, such as youth mental health. This variety provided a range of topics and flexibility for sessions that were participant nominated and participant led.

The feedback on the next steps aligned well with the goals of the CHRISA Unconference to enable connections and networking as well as discussion of issues to come up with recommendations. However, participants appeared to lack a willingness, confidence and/or understanding of the aspects of the issue for them to take on a leadership role. Thus, more planning to enable ‘thinking outside the box’ is needed for participants to generate novel and/or innovative ideas and multifaceted solutions for the next steps.

Sometimes, small, incremental changes that produce the most valuable outcomes tend to be overshadowed by a tendency to fixate on a single seemingly large problem with no obvious solution. Additionally, there appeared to be an expectation that the organizers of the Unconference would come up with the next steps and tell them what to do. Thus, in future events, it is imperative to develop leadership, enable participant‐led initiatives to ensue, provide training for participants and facilitators to decouple system thinking into smaller ‘bite‐size’ pieces and provide additional facilitator training for encouraging and ‘breaking’ down the propensity of macro solutions.

Although discussions were productive, the advancement of conversations could have been improved with semi‐structured or more focused discussions. This would allow groups to settle on a matter to discuss sooner, leaving more time for the identification of multifaceted solutions and input from more individuals. That said, there is a fine line between too much structure resulting in the ‘same old’ discussions and unstructured discussions resulting in creative, innovative ‘new ways’ and approaches to curing the crisis plaguing healthcare systems. An intermediate approach would be to start with a semi‐structured discussions that gradually moved to unstructured discussions. For example, an assessment of the pros and cons of Budd et al.'s 10 rules to examine the CHRISA Unconference for what worked and what could be improved would benefit the next iteration of the Unconference format [9].

### 4.1 Where to Go From Here

The CHRISA Unconference offered a creative platform for various groups and organizations to connect and discuss crucial healthcare system issues that are important to them and to propose ‘new ways and ideas’ for addressing the factors contributing to the challenges in healthcare crisis. Instead of discipline‐specific keynotes, the Unconference featured keynote presentations from individuals with direct experiences in the healthcare system and experts in design thinking and innovation to encourage ‘thinking outside the box’. This distinctive approach fostered networking and discussions as well as ‘planted the seed’ for ongoing collaboration for generating incremental, innovative solutions. Therefore, in future planning of an Unconference, it is important to consider timing or possibly hybrid options to ensure adequate representation from rural communities and to manage participant expectations about the potential benefits of unstructured discussions and solution generation.

Inclusion should also include inviting elected officials and decision makers. This would allow these individuals a unique opportunity to hear concerns, ideas and solutions from those ‘on the ground’. Their attendance would also be a powerful stepping stone to securing their input and support, which is necessary for most large, incremental or small‐scale solutions for healthcare systems. Lastly, elected officials and decision makers can provide information and explain the context that attendees and leaders may not otherwise consider or have access to pertinent details.

### 4.2 Recommendations

Building on the lessons learned from the CHRISA Unconference, we propose several recommendations for organizing future Unconference.

(1) *Focused Topics*: Organizers should consider selecting a few key themes or topics to guide the breakout sessions and discussions. This will help ensure that each session addresses specific challenges and allows for deeper exploration and actionable takeaways.
(2) *Additional Training*: Organizers should consider providing advanced training for ‘design thinking’ techniques to start from scratch, instead of what we know, to create openness to many different types of possibilities and solutions and to ‘decouple’ the pieces into smaller, ‘doable’ parts.
(3) *Involvement of Decision Makers*: Inviting elected officials and decision makers to participate in the Unconference can facilitate greater support and implementation of innovative ideas and solutions discussed during the event.
(4) *Rural Perspective*: Considering the rural perspective is essential for healthcare initiatives in southern Alberta. Timing the Unconference to avoid conflicts with important rural activities, such as seeding, can increase participation from rural communities.
(5) *Coordinated Discussions*: Providing some level of coordination or guidance for breakout sessions to ensure that each group has clear objectives and actionable outcomes will enhance the overall effectiveness of the Unconference.
(6) *Networking Opportunities*: Organizing networking opportunities, such as speed networking sessions or themed meet‐and‐greet events, can facilitate meaningful connections among participants and foster potential collaborations beyond the Unconference.

## 5 Conclusion

The CHRISA Unconference achieved its goals of fostering collaboration across diverse disciplines and organizations while engaging community partners to address healthcare challenges and propose new ideas and potential solutions. The rich and diverse knowledge base created during the Unconference is a foundation for future initiatives and collaborations in healthcare research, practice and change. To enable continued discourse for attendees, CHRISA will make this report available to attendees and distribute it to key partners in research, practice, policy and programming. CHRISA will continue to provide updates via newsletters and/or social media to support ongoing networking and collaborations. Implementing the recommendations gathered from the participant feedback will enhance future Unconferences and contribute to meaningful conversations to produce sustainable change in the healthcare system.

## Author Contributions

**Brenda M. Y. Leung:** conceptualization, planning and organization, evaluation and session notes appraisal, writing–original draft preparation, supervision, funding acquisition. **Helen Kelley:** planning and organization, writing–original draft preparation. **Angie Nikoleychuk:** writing–original draft preparation. **Gabrielle Kirk:** planning and organization, evaluation and session notes appraisal, writing–original draft preparation. **Fatemeh Salehi Shahrabi:** planning and organization, evaluation and session notes appraisal, writing–original draft preparation. **Victoria Hecker:** writing–review and editing. **Nolan Schaaf:** writing–review and editing. All authors have read and agreed to the published version of the manuscript.

## Conflicts of Interest

The authors declare no conflicts of interest.

## Data Availability

Data are not available for external use.

## 1

CHRISA Unconference Agenda

***Day 1 Breakout session topics***

- Falling through the cracks
- Social prescribing
- Health status and quality of life of frontline workers
- Supporting the independence of older adults through nutritional risk screening
- The role of intersectionality and access to health services
- How to create client‐centred solutions for healthcare
- How Library programmes help address community health challenges
- System integration—How do we build and maintain meaningful connections?
- Patient education
- Health at work—muscular skeletal demands
- Smoking and vaping in school environments
- Positive psychology and team building
- Population health and health equity
- Healthcare privatization
- Challenges faced by complex and chronic pain patients
- Mental health and addictions

***Day 2 Selected topics for breakout sessions***

- System integration in additions and mental health
- The role of intersectional and access to health services
- Rural health access
- Complex and chronic pain patients
- Income and food security
- Indigenous health
- Community‐based integration of services
